# Genetic Divergence in Domesticated and Non-Domesticated Gene Regions of Barley Chromosomes

**DOI:** 10.1371/journal.pone.0121106

**Published:** 2015-03-26

**Authors:** Songxian Yan, Dongfa Sun, Genlou Sun

**Affiliations:** 1 College of Plant Science and Technology, Huazhong Agricultural University, Wuhan, 430070, China; 2 Department of Biology, Saint Mary’s University, Halifax, Nova Scotia, B3H 3C3, Canada; Murdoch University, AUSTRALIA

## Abstract

Little is known about the genetic divergence in the chromosomal regions with domesticated and non-domesticated genes. The objective of our study is to examine the effect of natural selection on shaping genetic diversity of chromosome region with domesticated and non-domesticated genes in barley using 110 SSR markers. Comparison of the genetic diversity loss between wild and cultivated barley for each chromosome showed that chromosome 5H had the highest divergence of 35.29%, followed by 3H, 7H, 4H, 2H, 6H. Diversity ratio was calculated as (diversity of wild type – diversity of cultivated type)/diversity of wild type×100%. It was found that diversity ratios of the domesticated regions on 5H, 1H and 7H were higher than those of non-domesticated regions. Diversity ratio of the domesticated region on 2H and 4H is similar to that of non-domesticated region. However, diversity ratio of the domesticated region on 3H is lower than that of non-domesticated region. Averaged diversity among six chromosomes in domesticated region was 33.73% difference between wild and cultivated barley, and was 27.56% difference in the non-domesticated region. The outcome of this study advances our understanding of the evolution of crop chromosomes.

## Introduction

Domestication is a complex evolutionary process involving interaction between humans and the plants they used [1]. Selection has led to morphological and physiological changes between domesticated taxa and their wild ancestors [2], and shaped the genomes of all living creatures in earth, including domesticated plants and animals. Darwin clearly believed that breeds were formed by both natural and artificial selections, “The key (to domestic breeding) is man's power to accumulative selection: nature gives successive variations; man adds them up in certain directions useful to him” [3]. When selective pressure acts on individuals, it leads to the changes of genetic content in the population [4].

Two types of selection might impose on a species during domestication. Positive selection (purifying or directional selection), which refers to the selection process through it a particular phenotype (or genotype) is favored in a given environment, and leads to an increase of allelic frequency in a population [5,6]. Balancing selection, which refers to the selective process through it multiple alleles are selected, preserves the genetic diversity in a population [6,7]. Balancing selection is often observed when heterozygous individuals have a competitive advantage [6]. A study on domesticated cattle has identified the genomic regions which are potentially linked to purifying or balancing selection, and enhanced our understanding of the effect of natural and artificial selections on shaping the genetic diversity of cattle populations [6]. It is possible to identify chromosomal regions which were involved in adaptive divergence by comparing relative levels of differentiation among large numbers of unlinked markers [8], and determine the extent to which selection is acting across the genome [9]. It has showed that intense directional selection dramatically reduced allelic diversity, at both the targeted and linked neutral loci [10,11]. *Drosophila* and human geneticists have identified genomic regions which may have experienced selection or a “selective sweep” [12–14]. It has been reported that the SSRs associated with selective traits as grain weight are perhaps subjected to selection and displayed reduced genetic diversity [15, 19].

It has showed that SSRs are non-randomly distributed across protein-coding regions, UTRs and introns. The SSRs within genes have been subjected to stronger selective pressure than those in other genomic regions, and thus the SSRs can be used for evaluating the effect of selection [16]. Natural selection may be the major evolutionary force causing adaptive genetic divergence. In addition, natural selection is a major force causing differentiation of both coding and noncoding SSRs by micro- and macro- evolutionary processes [17,18]. By comparing the differences in the genic fraction among the types of microsatellite motifs present and their level of polymorphism, a better understanding of the different selection pressure in the genome will be gained. Barley (*Hordeum vulgare* L.) is an important crop and has long been used for food and feed [20]. Cultivated barley is domesticated diploid species (2n = 14) from its wild progenitor *Hordeum vulgare* ssp. *spontaneum* [21]. Barley has been used as an model for genetic and physiological studies in the last century [22,23].

Comparison of divergence between wild and domesticated accessions can reveal the effect of selection in species domestication. The wild accessions offer original chromosome diversity, and domesticated accessions have experienced selective sweeps for both adaptation and agronomic performance. Natural environments selected for resistance to stress, while the farmers selected for agronomic performances, palatability, nutritional and other uses [24]. Genetic changes of major agronomic traits are the base of barley origin and domestication. In the process of barley domestication, three key traits, non-brittle rachis, six-rowed spike and naked caryopsis, were involved [25]. Other domesticated traits such as reduced dormancy, reduced vernalization requirement and photoperiod insensitivity have been well studied, and controlled by the genes of *btr1* and *btr2*, *vrs1*, *nud*, QTLs (*SD1* and *SD2*), *sgh1* or *Vrn-H2 (sgh2* or *Vrn-H1* and *sgh3* or *Vrn-H3*), *ppd-H1* and *ppd-H2*, respectively [26].

The Near East Fertile Crescent has been considered as a major center where barley was domesticated [27]. However, the Himalayas, Tibet, Eritrea, Ethiopia, and Morocco regions were considered as centers of diversification of cultivated barley [28–32]. It has been speculated that barley was domesticated more than once: one within the Fertile Crescent and second one 1,500–3,000 km farther east thatcontributed to diversity in barley from Central Asia to the Far East [33]. The wild barley germplasm from origin central was elite and diversiform, and some regions of Israel, Jordan and Turkey have many specific types of wild barley accessions [21,34,35], while the chromosome 2H of some Qinghai-Tibetan wild barley accessions and other parts of China landraces has many unique alleles [36].

However, little is known about the genetic divergence in the chromosomal regions with domesticated and non-domesticated genes. The objective of our study is to examine the genetic diversity in barley chromosomal regions with domesticated and non-domesticated genes using SSR markers. The outcome of this study will enhance our understanding of the evolution of barley chromosomes associated with barley domestication.

## Materials and Methods

### 2.1 Plant Materials

A total of 117 barley accessions were used in this study including 97 wild barley accessions and 20 domesticated accessions (S1 Table). The materials used in this study were provided by the USDA (the United States Department of Agriculture) and the Huazhong Agricultural University barley germplasm collection [30].

### 2.2 DNA extraction and SSR

The seeds were planted in pots with sand-peat mixture and maintained in a greenhouse. The DNA was extracted from young freeze-dried leaf tissue using the cetyltrimethylammonium bromide (CTAB) method of Stein et al. [37]. The quality of DNA was checked using 0.8% agarose gel electrophoresis, and the DNA concentration was measured using spectrophotometer [38], then the concentration of samples was adjusted and standardized to 20 ng/ μL in a TE buffer.

SSR markers were synthesized based on sequence information from the GrainGenes database(http://wheat.pw.usda.gov/GG2/index.shtml). Polymerase chain reaction (PCR) was carried out in a final volume of 15 μL, containing 3μL of the 20ng/μL genomic DNA, 1.5μL of 10× PCR buffer (with 15 mM Mg^2+^), 0.3 μL of 10 mM dNTP mixture, 2.0μL of a 2.5μM solution of the forward and reverse primers, and 0.6 units of TaqDNA polymerase (TakaRa Biotechnology, Dalian, China). DNA amplifications were performed in a thermocycler using the following touchdown PCR protocol: 1 cycle of 3 min at 94°C, followed by 10 cycles 94°C for 30 sec, 30 sec at 60°C (decreasing 1°C per cycle), 45 sec at 72°C, and additional 25 cycles of 30 sec at 94°C, 30 sec at 50°C, 45 sec at 72°C. The reaction ended with a 5 min extension at 72°C. PCR product was separated on 6% denaturing polyacrylamide gel and visualized using silver staining [38].

A total of 260 barley SSRs were screened for polymorphism among two wild and two domesticated barley accessions (the materials of HS29, HS57, HS101 and HS111), and the 111 SSRs that generated clearly expected alleles were used to analyze the 117 barley accessions.

### 2.3 Data analysis

Microsatellite data were scored for each individual, and the pattern amplified by microsatellite primers were scored as 1 (present) and 0 (absent). The data were analyzed using POPGENE version 1.32 [39]. The gene diversity, which is equivalent to the proportion of loci heterozygous per individual under Hardy-Weinberg expectations (expected heterozygosity), was calculated by the unbiased method of Nei [40] considering sample sizes [41].

In order to test effect of selection pressure on genetic diversity of domesticated gene region and non-domesticated gene region, we searched barley linkage mapping, and found that nine domesticated genes associated with six important agronomic traits (Table 1) on six chromosomes(1H, 2H, 3H, 4H, 5H, 7H) [26]. The SSR markers on each chromosome were then divided into two regions, within domesticated gene regions and without domesticated gene regions. The SSR markers within domesticated gene region were divided used the AMOVA method of Arlequin ver 3.5 [42]. We figured out the positions of domesticated genes and SSR markers on each chromosome from the GrainGenes database (http://wheat.pw.usda.gov/GG2/index.shtml) and other reports [43,44], and the markers near the gene position were considered within the domesticated gene if the calculated regions’ P-value was significant (P-value < 0.05) different from the non-domesticated region on the same chromosome. Diversity ratio was calculated using the formula: (diversity of wild type—diversity of cultivated type)/diversity of wild type×100%.

**Table 1:** AMOVA test showed significant difference of genetic diversity between domesticated regions and non-domesticated regions*.

Chromosome	Domesticated traits	Associated genes	Markers within domesticated region	P-value
1H	photoperiod insensitivity	*Ppd-H2*	GBM1272, HvHvA1, Bmag382	0.036
2H	two or six rowed spike	*Vrs1/vrs1*	Hv5s	0.037
	photoperiod insensitivity	*Ppd-H1*	GBM5018, HVM36	
3H	non-brittle rachis	*btr1* and *btr2*	Bmac67, GBM1413	0.014
4H	reduced vernalization	*sgh1*	Hvm67, GBM1220, Bmag138	0.033
5H	dormancy	QTL (*SD1*)	Bmag357, GBM1399	0.001
		QTL (*SD2*)	GBM1164	
7H	hulled or naked caryopsis	*Nud/nud*	Bmag746, GBM1359	0.002
	other domesticated genes or loci		GBM1456, HVM51	

Non-domesticated regions*: the regions on the same chromosome except domesticated regions.

## Results

### 3.1 SSR polymorphism on barley chromosomes

In this study, the expected heterozygosity of 111 SSR markers on the seven barley chromosomes were calculated (data not shown). On the chromosome 1H, the highest diversity for all 117 barley accessions was 0.927 (Bmag345), and the lowest was 0.085(GBM1278). The highest and lowest diversity on the chromosome 2H was 0.899 (EBmag793) and 0.324 (GBM5018), respectively. The genetic diversity ranged from 0 (Bmag23) to 0.935 (Bmac129) on 3H, from 0.067 (HVM77) to 0.912 (EBmac635) on 4H, from 0 (GBM1227) to 0.905 (Bmag113d) on 5H, from 0 (Bmac251) to 0.891 (Bmac18) on 6H, and from 0.096 (GBM1456) to 0.906 (Bmag7) on 7H.

For wild barley, the highest averaged diversity of 0.799 was observed on 2H, and lowest value (0.528) was observed on the chromosome 6H. For cultivated barley, the highest averaged diversity was 0.583 for 2H, and lowest diversity was 0.385 for 5H. The level of divergence between wild and cultivated barley for each chromosome was compared. Chromosome 5H had the highest divergence of 35.29% (from 0.596 to 0.385), followed by 3H, 7H, 4H, 2H, 6H and the lowest between wild and cultivated barley was 1H with 22.26% (from 0.734 to 0.571).

### 3.2 Gene diversity of domesticated gene region and non-domesticated region calculated by AMOVA method

**Fig 1:**
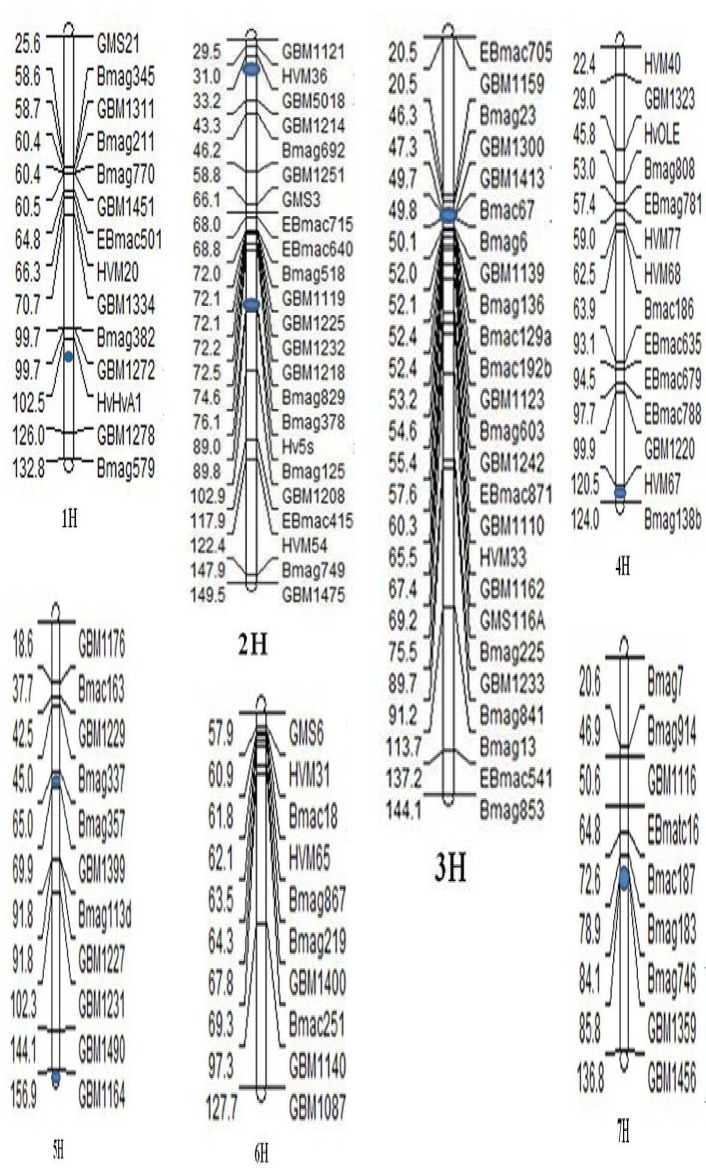
The SSR markers in domesticated regions and non-domesticated regions divided based on the genetic distance (cM). The location of SSR marker in each linkage group is mainly based on Varshney et al. (2007) [43]. The dot on the chromosome represented the position of domesticated genes: *Ppd-H2* gene on chromosome 1H, *Ppd-H1* gene (top) and *Vrs1* gene (bottom) on chromosome 2H, *btr1* and *btr2* genes (linked tightly) on 3H, *Sgh1* gene on the 4H, main QTLs (*SD1*, top and *SD2*, bottom) on chromosome 5H, and *nud* gene on the chromosome 7H, No domesticated gene on 6H chromosome.

The domesticated gene regions were defined as the chromosome fragments surrounding the domesticated genes. We have figured out nine domesticated gene positions on six chromosomes based on previous published reports [26, 45–49]. The SSR markers and the domesticated gene on each chromosome were showed in Fig. 1. Based on position of SSR on each chromosome, first, we selected a relative large region on a chromosome with many SSR molecular markers flanking the domesticated gene; then calculated the gene diversity within and outside this region for each chromosome, and compared diversity between them. If the diversity was not significant difference between two regions on each chromosome, we narrowed down the domesticated gene region, and reexamined difference until P-value was significant (P value < 0.05). For example, we classified the SSR markers on the chromosome 3H into two groups, one was the domesticated *btr1* and *btr2* genes and its nearby region, the other was rest of region on the chromosome. When the domesticated gene region contained three markers (Bmac67, GBM1413, and Bmag6), the significant P-value between domesticated and non-domesticated regions of this chromosome was 0.263. Then we narrowed down the domesticated region to include only Bmac67 and GBM1413 markers, and the P-value reached significant with 0.014< 0.05, so we consider that the *btr1* and *btr2* genes region contained two markers of Bmac67 and GBM1413. The results were presented in the table 1.

Among the 16 SSR markers on chromosome 1H, genetic diversities between the domesticated region of short-day flowing time *Ppd-H2* gene [45,46] and non-domesticated region were compared. The region of *Ppd-H2* gene with three markers (GBM1272, HvHvA1, Bmag382) displayed a significant difference in genetic diversity from the outside region (P = 0.036) (Table 1). On the chromosome 2H, Hv5s was close to row spike-types *Vrs1/vrs1* gene [50] and GBM5018 and HVM36 were within the long-day flowering time *Ppd-H1* gene region. The non-brittle rachis *btr* genes region included two markers (Bmac67 and GBM1413) on the 3H. Similarly, there was three SSR markers associated with the vernalization gene *sgh1* on the 4H. It has been known that two main QTLs (*SD1* and *SD2*) controlled the seed dormancy on the chromosome 5H, and three markers (Bmag357, GBM1399 and GBM1164) were close to them, respectively. There were four SSR markers within the domesticated regions of 7H, two markers (Bmag746 and GBM1359) within hulled/naked gene *Nud/nud* region, and the GBM1456 and HVM51 within other domesticated genes. It was noted that only if the two markers GBM1456 and HVM51 were included, genetic diversity in 7H domesticated region was significantly different from that in non-domesticated region (P = 0.002).

Based on Table 1, we divided the SSR markers on each chromosome into two groups, domesticated gene region and non-domesticated gene region group. Genetic diversity of these two regions on each chromosome was compared (Table 2). The level of genetic diversity change between wild and cultivated barley accessions on the domesticated region and non-domesticated region was measured as diversity ratio and given in Table 2. The diversity ratios of the domesticated regions on 1H, 5H and 7H were higher than those of non-domesticated regions, respectively. The diversity ratios of the domesticated regions on 2H and 4H were similar to that of non-domesticated regions, respectively. However, diversity ratio of the domesticated region on 3H was lower than that of non-domesticated region. The diversity within domesticated gene region of chromosome 5H had the highest diversity ratio (52.06%), followed by domesticated gene region on chromosome 7H, 4H, 1H and 2H. The domesticated region on 3H had smallest diversity ratio of -18.77%. However, the diversity of non-domesticated gene region on chromosome 3H had the highest diversity ratio of 34.91%, followed by 4H, 5H, 7H, 2H and 1H (19.30%) (Table 2). The highest difference (21.53%) of diversity ratio between domesticated region and non-domesticated region was observed on chromosome 5H, followed by 7H, 1H, 2H, 4H and 3H.

**Table 2:** Genetic diversity and diversity ratio in domesticated and non-domesticated gene regions of barley chromosomes.

Chromosome	Wild types	Cultivated types	Diversity Ratio (%)*
1H (domesticated region)	0.613	0.379	38.24
1H (undomesticated region)	0.762	0.615	19.30
2H (domesticated region)	0.542	0.407	24.83
2H (undomesticated region)	0.842	0.635	24.61
3H (domesticated region)	0.515	0.611	-18.77
3H (undomesticated region)	0.744	0.485	34.91
4H (domesticated region)	0.757	0.519	31.44
4H (undomesticated region)	0.811	0.550	32.22
5H (domesticated region)	0.482	0.231	52.06
5H (undomesticated region)	0.638	0.443	30.53
7H (domesticated region)	0.464	0.277	40.27
7H (undomesticated region)	0.815	0.603	26.04
Average domesticated region	0.558	0.370	33.73
Average undomesticated region	0.741	0.536	27.56

*: diversity ratio = (diversity of wild type—diversity of cultivated type)/diversity of wild type×100%

## Discussion

### 4.1 Genetic variation of each chromosome

Previous studies have demonstrated that SSRs markers displayed a very high degree of polymorphism in both wild barley and landrace accessions [18,36]. Our results indicated that the chromosome 2H has the highest level of gene diversity (0.792) among the 7 chromosomes, which is in agreement with the study of Gong [36]. It was well known that the chromosome 2H contains many important genes for barley development and adaptation,such as row-type *vrs1*[50], earliness per se *eps2S* [51, 52], early maturity *Eam1*[51] and heading date *Ppd-H1*[45,46], which might keep chromosome 2H diversified in both wild and domesticated barley. We also found that the wild barley chromosome 6H has a relatively low diversity, which is consistent with Russell et al [53].

The highest divergence level of 5H between wild barley and cultivated barley was observed, and 3H also has a relatively higher level of divergence between wild barley and domesticated barley. It might be caused by selection during domestication since chromosome 5H contains many domesticated genes or major QTLs such as *SD1*, *SD2* and *sgh2*. The two major QTL, *SD1* and *SD2*, located at different loci on 5H which determine seed dormancy [26, 47]. Vernalization gene *sgh2* also located on the 5H, which controls the vernalization together with other two genes of *Sgh1*(4H) and *sgh3*(7H) [26, 48]. It has been reported that the genetic differentiation is uneven across genome, and is greatest on linkage groups 5H and 2H between east and west wild barley populations of the Zagros Mountains and influenced by different environmental factors [54]. From wild progenitor to domesticated cultivars, the domesticated gene may be suffered from “domestication bottleneck” [55]. Gene diversity decrease on chromosome 3H in cultivated barley might be attributed to the existence of *btr1* and *btr2* on chromosome 3HS and other domesticated genes or loci. The tightly linked recessive *btr1* and *btr2* were the most important domestication related genes which determine the non-brittle rachis traits, and were independently established by natural mutations from wild types of *Btr1* and *Btr2*, respectively [26,49].

### 4.2 Selection pressure on domesticated and non-domesticated chromosomal regions

Crop species experienced strong selective pressure on genes controlling traits of agronomic importance during their domestication [56], and the remaining genes retained evidence of a population bottleneck associated with domestication [57]. Comparison of diversity from domesticated and non-domesticated gene regions showed that different chromosomal regions had been subjected to diverse natural selection pressure. The diversity ratios of the domesticated regions on 5H, 1H and 7H were higher than those of non-domesticated regions. The reductions of variation resulting from strong selective pressure on particular loci have been also observed in genes associated with domestication or diversification phenotypes [10,58]. The chromosome 5H have several domesticated genes and adaptive SSRs such as GMS61, GMS1 and EBmac824, and natural selection pressure may strongly act upon these regions by directional selections [59]. It is well known that many important QTL or genes controlling number of seeds per spike [60], disease resistance [61], kernel weight and the number of spikelets per ear [62] have been detected on chromosome 1H, respectively. Chromosome 7H also contains many important domestication related genes such as naked gene *nud* and vernalization gene *sgh3*. The *nud* gene controls naked seed [26,63]. Our results demonstrated that domesticated gene regions have been under strong positive selection pressure [6] which could markedly reduce recombination rates and genetic diversities [10,14,64]. Kim et al. [65] also found that more than 40 genomic regions were under selection on several U.S. Holstein cattle chromosomes, and many of these selected regions were associated to important trait loci controlling milk, fat, and protein. Other factors such as recombination rate, population size, population structure, and breeding systems also affect the genetic diversity during barley domestication [6, 9].

Our data showed that genetic diversity in non-domesticated region of chromosome 3H was dramatically reduced in the domesticated accessions, suggesting that this region might be subjected to a relatively strong positive selection pressure. The non-domesticated chromosomal region that we classified on 3H contained some other important gene such as the *sd* (dwarfing) gene [53], and several major QTLs controlling thousand grain weight [66], plant height and spike length [60], disease resistance traits [67] and chlorophyll enzyme biosynthesis [68]. During domestication, artificial selection for these genes or major QTL could cause divergence of non-domesticated region between wild and domesticated accessions. While diversity ratios of the domesticated region on 2H and 4H were similar to those of its non-domesticated regions, these two chromosomes may have suffered a balancing selection pressure between domesticated and its respective non-domesticated regions. It is certain that barley chromosome 2H is an important reservoir of molecular polymorphism [36], as the chromosome 2H of Tibetan barley landraces possess many unique alleles which may promote barley adaptation to diverse environments. It has been reported that the short arm of chromosome 4H had a significantly low single-nucleotide variants frequency, which might be caused by reduction in recombination frequency on this chromosome that was linked with recent breeding history or landmarks of barley domestication [69].

In this study difference of averaged diversities (between wild and cultivated populations) in domesticated regions among six chromosomes was 33.73%, and was 27.56% in non-domesticated regions. This might suggest that domesticated regions, in general, were under a positive selection pressure in the process of domestication which increased prevalence of advantageous traits [70]. The selection pressure on chromosomal 5H, 1H and 7H domesticated regions was relatively stronger than other regions, while the domesticated regions on 2H and 4H might suffer a moderate selection. In contrast, chromosome 3H might suffer a diverse selection pressure for domesticated region. It has also been shown that some regions of human genomes might have been subjected to positive selection, and the effects of positive selection may be more pronounced on the X chromosome than on the autosomes [14].

Chromosomal evolution included a continuum of molecular-based events of greatly varied scope which forced by modification, acquisition, deletion, and/or rearrangement of genetic material [71]. Knowledge of diversity changes on different chromosomal regions between wild and cultivated barley provides important information for our understanding of the barley chromosomal evolution, which is the fundamental to barley origin, survival, and adaptation. Moreover, some chromosomal regions or loci may be specific based on the variation of diversity, it could be a potential source for exploiting and utilizing novel barley germplasms in the future crop improvement.

We understand that the methods used in this study have some limitations. The way to define chromosomal region with domesticated genes and non-domesticated gene is really loose. It cannot be ruled out that there are no domestication genes in non-domesticated gene region due to the marker coverage. Clearly, all the defined chromosomal regions have different lengths and are too large segment that contains too many genes either for domestication or not. The straight way is to find functional SSR markers from published domestication genes and those genome SSR markers with known linkage position in future study.

In conclusion, our study showed that difference in averaged diversity of domesticated regions between wild and cultivated barley populations was higher than that of non-domesticated chromosomal regions. However, this study had focused only on selection at different barley chromosomal regions during barely domestication. The lack of enough polymorphic markers prevents us to infer how large regions of domesticated gene on each chromosome are affected by natural selection. Further research with dense SSR or SNP markers is needed to understand the selection impacts.

## Supporting Information

S1 Table The code, accession number, origin and characteristic of 117 barley accessions used in this study. (DOCX)
