# Supplementary material for: Genetic Divergence in Domesticated and Non-Domesticated Gene Regions of Barley Chromosomes
Source: PLoS One. 2015 Mar 26;10(3):e0121106. doi: 10.1371/journal.pone.0121106 (PMC4374956; doi:10.1371/journal.pone.0121106)
Supplement: S1 Table — (DOCX) [file pone.0121106.s001.docx]

Table S1. The code, accession number, origin and characteristic of 117 barley accessions

used in this study.

|  |  | |  |  |  | |  | |  |  |
| --- | --- | --- | --- | --- | --- | --- | --- | --- | --- | --- |
| Code | Accession Number | | Origin | Characteristic | Code | | Accession Number | | Origin | Characteristic |
| HS1 | | PI212305 | Afghanistan | wild，two-row | | HS60 | PI466618 | Iran | | wild，two-row |
| HS2 | | PI212306 | Afghanistan | wild，two-row | | HS63 | PI554426 | Turkey | | wild，two-row |
| HS3 | | PI219796 | Iraq | wild，two-row | | HS64 | PI466632 | Iran | | wild，two-row |
| HS4 | | PI220664 | Afghanistan | wild，two-row | | HS65 | PI466699 | Iran | | wild，two-row |
| HS5 | | PI227019 | Iran | wild，two-row | | HS66 | PI554428 | Turkey | | wild，two-row |
| HS6 | | PI235639 | Germany | wild，two-row | | HS67 | PI559556 | Turkey | | wild，two-row |
| HS7 | | PI236386 | Syria | wild，two-row | | HS68 | PI662052 | Tajikistan | | wild，two-row |
| HS8 | | PI244772 | Pakistan | wild，two-row | | HS69 | PI662080 | Tajikistan | | wild，two-row |
| HS9 | | PI244774 | Afghanistan | wild，two-row | | HS70 | PI662109 | Tajikistan | | wild，two-row |
| HS10 | | PI244776 | Afghanistan | wild，two-row | | HS71 | PI662118 | Tajikistan | | wild，two-row |
| HS11 | | PI244777 | Afghanistan | wild，two-row | | HS72 | PI662138 | Turkey | | wild，two-row |
| HS12 | | PI245739 | Turkey | wild，two-row | | HS73 | PI662158 | Turkey | | wild，two-row |
| HS13 | | PI253933 | Iraq | wild，two-row | | HS74 | PI662170 | Turkey | | wild，two-row |
| HS14 | | PI254894 | Iraq | wild，two-row | | HS75 | PI662178 | Turkey | | wild，two-row |
| HS15 | | PI268243 | Iran | wild，two-row | | HS76 | PI662188 | Turkey | | wild，two-row |
| HS16 | | PI283422 | Sweden | wild，two-row | | HS77 | PI662204 | Turkey | | wild，two-row |
| HS17 | | PI283423 | Former Soviet Union | wild，two-row | | HS78 | PI662214 | Turkey | | wild，two-row |
| HS18 | | PI284752 | Israel | wild，two-row | | HS79 | PI662218 | Turkey | | wild，two-row |
| HS19 | | PI293411 | Tajikistan | wild，two-row | | HS80 | 42 | Sichuan, China | | landrace, naked, six-row |
| HS20 | | PI293412 | Tajikistan | wild，two-row | | HS81 | 43 | Sichuan, China | | landrace, naked, six-row |
| HS21 | | PI296413 | Azerbaijan | wild，two-row | | HS82 | 45 | Sichuan, China | | landrace, naked, six-row |
| HS22 | | PI293414 | Azerbaijan | wild，two-row | | HS83 | 46 | Sichuan, China | | landrace, naked, six-row |
| HS23 | | PI296792 | Israel | wild，two-row | | HS84 | 177 | Hunan, China | | landrace, naked, six-row |
| HS24 | | PI296849 | Israel | wild，two-row | | HS85 | 87 | Henan, China | | landrace, hulled, six-row |
| HS25 | | PI296862 | Israel | wild，two-row | | HS86 | 91 | Sichuan, China | | landrace, hulled, six-row |
| HS26 | | PI296878 | Israel | wild，two-row | | HS87 | 274 | Sichuan, China | | landrace, hulled, six-row |
| HS27 | | PI296908 | Israel | wild，two-row | | HS88 | 276 | Shandong, China | | landrace, hulled, six-row |
| HS28 | | PI354948 | Israel | wild，two-row | | HS89 | 384 | Zhejiang, China | | landrace, hulled, six-row |
| HS29 | | PI356061 | Ethiopia | wild，two-row | | HS90 | 56 | Sichuan, China | | landrace, naked, two-row |
| HS30 | | PI356209 | Ethiopia | wild，two-row | | HS91 | 248 | Sichuan, China | | landrace, naked, two-row |
| HS31 | | PI391100 | Israel | wild，two-row | | HS92 | 252 | Sichuan, China | | landrace, naked, two-row |
| HS32 | | PI401368 | Iran | wild，two-row | | HS93 | 279 | Sichuan, China | | landrace, naked, two-row |
| HS33 | | PI401371 | Iran | wild，two-row | | HS94 | 353 | Sichuan, China | | landrace, naked, two-row |
| HS34 | | PI420911 | Jordan | wild，two-row | | HS95 | 65 | Sichuan, China | | landrace, hulled, two-row |
| HS35 | | PI420912 | Jordan | wild，two-row | | HS96 | 381 | Sichuan, China | | landrace, hulled, two-row |
| HS36 | | PI420913 | Jordan | wild，two-row | | HS97 | 382 | Zhejiang, China | | landrace, hulled, two-row |
| HS37 | | PI420915 | Jordan | wild，two-row | | HS98 | 383 | Zhejiang, China | | landrace, hulled, two-row |
| HS38 | | PI420916 | Jordan | wild，two-row | | HS99 | 385 | Zhejiang, China | | landrace, hulled, two-row |
| HS39 | | PI420917 | Jordan | wild，two-row | | HS100 | 1 | Tibet, China | | wild, two-row |
| HS40 | | PI466040 | Syria | wild，two-row | | HS101 | 3 | Tibet, China | | wild, two-row |
| HS41 | | PI466048 | Syria | wild，two-row | | HS102 | 4 | Tibet, China | | wild, two-row |
| HS42 | | PI466060 | Syria | wild，two-row | | HS103 | 13 | Tibet, China | | wild, two-row |
| HS43 | | PI466086 | Syria | wild，two-row | | HS104 | 18 | Tibet, China | | wild, two-row |
| HS44 | | PI466118 | Syria | wild，two-row | | HS105 | 22 | Tibet, China | | wild, two-row |
| HS45 | | PI466130 | Syria | wild，two-row | | HS106 | 23 | Tibet, China | | wild, two-row |
| HS46 | | PI466178 | Syria | wild，two-row | | HS107 | 26 | Tibet, China | | wild, two-row |
| HS47 | | PI466206 | Syria | wild，two-row | | HS108 | 27 | Tibet, China | | wild, two-row |
| HS48 | | PI466238 | Syria | wild，two-row | | HS109 | 28 | Tibet, China | | wild, two-row |
| HS49 | | PI466249 | Lebanon | wild，two-row | | HS110 | 2 | Tibet, China | | wild, six-row |
| HS50 | | PI466256 | Lebanon | wild，two-row | | HS111 | 5 | Tibet, China | | wild, six-row |
| HS51 | | PI466264 | Lebanon | wild，two-row | | HS112 | 6 | Tibet, China | | wild, six-row |
| HS52 | | PI466296 | Israel | wild，two-row | | HS113 | 7 | Tibet, China | | wild, six-row |
| HS53 | | PI466328 | Israel | wild，two-row | | HS114 | 9 | Tibet, China | | wild, six-row |
| HS54 | | PI466388 | Israel | wild，two-row | | HS115 | 10 | Tibet, China | | wild, six-row |
| HS55 | | PI466498 | Israel | wild，two-row | | HS116 | 11 | Tibet, China | | wild, six-row |
| HS56 | | PI466524 | Israel | wild，two-row | | HS117 | 14 | Tibet, China | | wild, six-row |
| HS57 | | PI466554 | Israel | wild，two-row | | HS118 | 15 | Tibet, China | | wild, six-row |
| HS58 | | PI466586 | Israel | wild，two-row | | HS119 | 19 | Tibet, China | | wild, six-row |
| HS59 | | PI466605 | Iran | wild，two-row | |  |  |  | |  |
